# Demographic Status and Genetic Tagging of Endangered Capercaillie in NW Spain

**DOI:** 10.1371/journal.pone.0099799

**Published:** 2014-06-13

**Authors:** María Morán-Luis, Alberto Fameli, Beatriz Blanco-Fontao, Alberto Fernández-Gil, Rolando Rodríguez-Muñoz, Mario Quevedo, Patricia Mirol, María-José Bañuelos

**Affiliations:** 1 Research Unit of Biodiversity (UO-PA-CSIC), University of Oviedo, Mieres, Spain; 2 Ecology Unit, Department of Biology of Organisms and Systems, University of Oviedo, Oviedo, Spain; 3 Group of Biodiversity and Conservation Genetics (GECOBI), Argentinian Museum of Natural Sciences ‘Bernardino Rivadavia’, Buenos Aires, Argentina; 4 Department of Conservation Biology, Doñana Biological Station (CSIC), Sevilla, Spain; 5 Centre for Ecology and Conservation, School of Biosciences, University of Exeter, Penryn, United Kingdom; University of Lleida, Spain

## Abstract

Counting rare and elusive animals and evaluating their demographic status, are fundamental yet challenging aspects of population ecology and conservation biology. We set out to estimate population size (*N_c_*), genetic effective population size (*N_e gen_*), sex ratio, and movements based on genetic tagging for the threatened Cantabrian capercaillie. We used 9 microsatellite loci to genotype 134 droppings collected at 34 display areas during the breeding season. Using genetic capture-mark-recapture, we estimated 93 individuals (*N_c_*, 95% CI: 70–116) in an area of about 500 km^2^, with sex ratio biased towards males (1∶1.6). Estimated *N_e gen_* (35.5) was 38% of *N_c_*, notably higher than the published average in wild populations. This capercaillie population is small and well within concern in terms of population viability. By genetic tagging, we detected mostly short movements; just a few males were recaptured between contiguous display areas. Non-invasive surveys of endangered populations have a great potential, yet adequate sample size and location are key to obtain reliable information on conservation status.

## Introduction

We deem populations as endangered when they are small, declining, or both; they are susceptible to environmental and demographic stochasticity, loss of genetic variability, and inbreeding depression [1], [2]. On top of that, we know that fragmentation often increases extinction risk, either directly due to lessened connectivity or indirectly due to Allee effects [3]. Then, it is important to know when a population is threatened, and its status needs to be soundly assessed.

**Figure 1:**
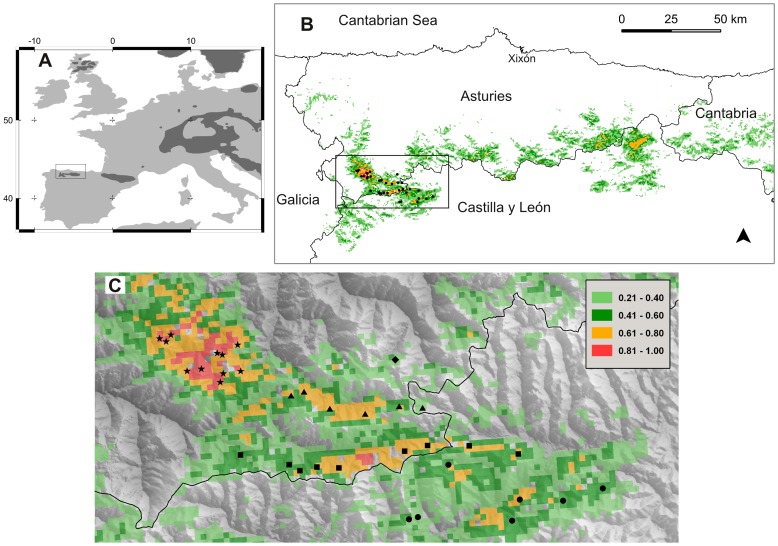
Distribution of Cantabrian capercaillie (*Tetrao urogallus cantabricus*) and location of the study area. A) Distribution of capercaillie in central and southwestern Europe (dark grey). B) Location of the study sites (filled dots) over an index of habitat suitability [31]. C) Detail of the study area; different symbols show display areas where samples were collected in spring 2009 in each of five watersheds: Muniellos (stars), Hermo (triangles), Degaña (squares), Leitariegos (diamonds) and Alto Sil (dots). The legend shows habitat suitability according to [31].

Cantabrian capercaillie (*Tetrao urogallus cantabricus*), a polygynous forest galliform found in the fragmented forest ecosystem of NW Spain (Figure 1), is one of the most endangered tetraonids [4]. Its range has declined steeply since the early 1980s [5], [6], and it has been isolated long enough from other capercaillie populations to be considered an Evolutionary Significant Unit [7]. The subspecies has additional biogeographical interest due to its location at the rear edge of the species range [8]. In the early 2000s, six hundred individuals were coarsely estimated in an area of occupancy of 1700 km^2^ [4]. Since then, unpublished data suggest that present capercaillie presence in eastern and central parts of the range (Figure 1B) is tenuous at best. However, these population estimates had been based on direct observations, whose accuracy is debatable and context-dependent (e.g. [9], [10]). Usually the bias is higher in rare and elusive species [11]. But even in more conspicuous species, direct counts entail potential biases for the more cryptic components of the population (e.g. [12], [13]). Capercaillie is a good example: in the breeding season, they gather at display areas, where dominant males display and court the smaller, less showy females [14]. If birds were displaying, an experienced observer might see or hear the dominant males, and perhaps some females. However, direct counts at display areas are not an appropriate method to detect females, which have a cryptic plumage and attend display areas for shorter periods [14], [15], or subordinate/non-breeding males [16]. Thus, direct counts at display areas typically underestimate real numbers in capercaillie [17]. Nonetheless, even with these coarse estimates, the population of Cantabrian capercaillie is small and declining.

In conservation biology it is relevant to know which proportion of the population contributes to reproduction. The demographic effective population size (*N_e_*) is roughly equivalent to the number of breeders in a given year [18], and is largely influenced by sex ratio and mating systems [19], [20]. In polygynous species with lekking behavior such as capercaillie, a few males monopolize mating [16], [21], reducing notably *N_e_* below the number of sexually mature adults in the population. Fluctuations in population size, variability in individual reproductive success, and unequal sex ratios are thus responsible for additional reductions of *N_e_* [18], [22], [23]. Ultimately, populations with low *N_e_* are more prone to extinction [24]. Several methods based on genetic data, have been developed to compute *N_e_*-related indices [25], [26]. One of them is the genetic effective population size (*N_e gen_*), defined as the number of breeding individuals in a theoretical population that would lose genetic diversity at the same rate as the real population being studied [27]. However, *N_e gen_* is not an easy parameter to estimate in the wild; localized breeding in continuous populations may be subjected to the effects of isolation by distance, affecting *N_e gen_* estimates and being sensitive to the geographical scale of the sampling [28].

Non-invasive census methods based on DNA analysis are now available, ‘capturing’ the genetic material instead of the individual itself [29], [30]. They increase capture probabilities while greatly reducing disturbances. Genetic estimates are based on the individual identification of materials left behind by the animal, like feces, feathers or hairs, a sort of molecular tagging (e.g. [31], [32]). Therefore individuals can be tracked, and specific methods have been developed to estimate abundance and effective population size, even from a single sampling session [33]. Last but not least, with an adequate sampling design, these methods allow to minimize potential biases due to differences in behavior and habitat use of the different components of the population [12].

We performed a genetic survey of Cantabrian capercaillies in a large portion of the extant distribution using multilocus genotyping of capercaillie droppings as individual molecular tagging. We aimed at getting information on abundance, sex ratio, effective population size, and movements to determine conservation status and inform management decisions.

## Methods

### Ethics statement

Cantabrian capercaillie is an endangered subspecies, and access is restricted to areas where spring display has been recorded. The environmental authorities of Asturias and Castilla y León granted the required permits for this study, which is solely based on non-intrusive sampling of droppings. The survey was specifically designed to minimize disturbance to capercaillie behavior. Each sampling session was carried out by two people, starting well after capercaillie displays were over. The study did not require ethical approval.

### Study area and field survey

We conducted our study in the western part of the Cantabrian Range, where capercaillie inhabits a mountainous landscape with highly fragmented forests [6], [34]. We surveyed some of the least disturbed forest patches, that combined higher habitat suitability and current capercaillie presence [35] (Figure 1B). However, there are several, recently described leks south of our study area, which were not covered by the aforementioned study of habitat suitability [36].

We defined five survey zones in the study area, essentially following the main sub-watersheds (Figure 1C, Table 1). All zones are in sites designated as EU Natura 2000 (Habitats Directive 92/43/CEE). In addition, three of them (Leitariegos, Hermo and Degaña) are also included in a regional park, and the other one (Muniellos) is a natural reserve where only a few visits are allowed per day along a designated foot path. The total study area was about 500 km^2^ with a forest cover of 45%.

**Table 1:** Minimum (*N_min_*) and estimated (*N_c_*) number of capercaillies in the study area.

Survey zone	N_min_	N_min female_: N_min male_	*N_c_* (95% CI)	N_c female_: N_c male_
Muniellos	15	7∶7 (1)	19 (15–26)	-
Hermo	7	2∶5 (0)	-	-
Degaña	17	6∶10 (1)	-	-
Leitariegos	4	1∶2 (1)	-	-
Alto Sil	13	4∶8 (1)	-	-
Total	56	20∶32 (4)	93 (70–116)	28∶44

Population size (Nc) was estimated both for the whole study area and for Muniellos, the best represented zone in the samples. Separate estimates for each sex (Nc male, Nc female) are indicated for the whole study area. Numbers in parentheses indicate individuals that could not be sexed.

Sampling took place from April to early June 2009, i.e. during the mating season of Cantabrian capercaillie. We searched for droppings in forest patches that included 52 previously known display areas (i.e. sites where one or more cocks consistently display for females [37]). Our survey included 71% of all known display areas in the study area with occupancy data since 2000. Each display area was surveyed during 2 to 3 hours by two people and each sample location was recorded with a GPS (±5m). We selected droppings based on their appearance (size, shape and content) and distance (25 m for those similar-looking samples). Droppings were stored into tubes with silica-gel and frozen at -20°C until DNA extraction. We performed a second survey after 2–3 weeks in those places where no sample could be found during the first visit.

### Laboratory procedures

We extracted genomic DNA, from a pool of 291 samples distributed throughout the study area (Figure 1C). Polymerase chain reaction (PCR) amplifications were conducted for 9 microsatellite loci, previously developed for *Tetrao urogallus* (TUD2, TUD4, TUD5, TUT1, TUT3), and *Tetrao tetrix* (TTD2, TTD6, BG10, BG15) [38]–[40]. PCR-amplifications were performed in 10 µl reactions mix containing 2 µl of extract DNA, 1x Taq buffer (750 mm Tris-HCl, 200 mM (NH_4_)_2_SO_4_, 0.1% (v/v) Tween 20), 3 mM MgCl_2_, 0.2 mM of each nucleotide, 4.2 pmoles of each primer, 0.108 µg/ µl de BSA and 0.335 units of DNA Taq polymerase (Fermentas). PCR conditions consisted of 3 minutes at 94°C plus 35 cycles of 45 seconds denaturing at 94°C, 45 seconds annealing at 54°C (for BG10 and BG15) or at 59°C (for the rest of the primers), 45 seconds extension at 72°C, and 5 minutes at 72°C for the final extension. We amplified microsatellite loci individually and negative controls were included in all amplification reactions. Extraction and amplification were performed in dedicated and separated. Extraction was confirmed by amplifying a single microsatellite (TUT1). When a sample did not render a positive result, a second PCR was performed. Samples that tested positive in one of the two independent PCRs were included in the analysis for the next step.

Each sample was amplified 2–7 times to minimize genotyping errors, following a modification of the multiple-tube approach [41], [42]. A consensus genotype was determined for each sample after a minimum of two independent positive PCRs for heterozygotes, and three for homozygotes.

To determine the sex of each identified individual, we used capercaillie-specific sex primers [43], derived from the chromosome-specific intron size difference in the CHD1 gene (located in bird sexual chromosomes). These primers produce sex-specific short fragments (about 200 bp), and perform well with degraded DNA samples, such as those coming from feces. Consensus genotypes were determined following the same criteria as for microsatellite loci, i.e. a minimum of two independent positive PCRs for heterozygotes (ZW, females) and three for homozygotes (ZZ, males).

Genotyping was performed in two laboratories (GECOBI Lab, Argentina and UMIB-Molecular Ecology Lab, Spain), using two different sequencing machines, MegaBace 1000 automated Sequencer (Argentina) and ABI Prism 3100 Genetic Analyzer (Spain). Microsatellites were genotyped in three post-PCR multiplexes, based on allele size ranges and fluorescent dyes. When genotyping is performed by several laboratories and/or platforms, calibration and standardizing of allele size designation is necessary [44], [45]. We standardized microsatellite scores with template DNA from samples containing the full range of alleles found in our study area. We established standardization rules following [45] recommendations. Sizing was double-blind checked, using two different software packages: MegaBACE Fragment Profiler 1.2 software (Amersham Biosciences) and GeneMarker v1.3 (Soft Genetics LLC). We then performed double-blind re-screening of all samples in both labs, to confirm standardization rules, and inconsistencies other than size shifts were also discussed. When required, new amplifications were performed and samples were re-screened again. If the inconsistency persisted, the locus was not considered in that sample.

### Genotyping data

We estimated the number of alleles observed per locus (A), observed and expected heterozygosities (H_o_ and H_e_ respectively), and deviations from Hardy-Weinberg equilibrium (HWE), using GENEPOP 4.2 [46].

The amount of target DNA available in fecal samples is often low, leading to an increase of genotyping errors [30], [41]. Genotyping errors (false alleles and allelic dropout) at each locus across PCRs were checked using GIMLET v.1.3.3 [47]. With the estimated error rates, we compared the results from independent PCRs and the associated consensus genotype for all the amplified samples, regardless they were included in the final dataset (i.e. 212 feces, see Results). We used MICRO-CHECKER 2.2 [48] to check for large allele dropout, stuttering and null alleles that can underestimate the number of individuals inflating the proportion of homozygotes. This software analyzes deviations from Hardy-Weinberg proportions using consensus genotypes of all different individuals identified, to detect loci with potential errors.

To check the power of the chosen microsatellite loci for identifying individuals, we calculated the probability that two individuals, drawn at random from the population, would share the same multilocus genotype (probability of identity, P_ID_; [51]), even if they are full siblings or have a high degree of kinship (P_ID-sib_, [51]). This probability depends on the number of selected loci used to construct the genotype, the variability of these loci, and the relatedness of individuals within the population. Both P_ID_ and P_ID-sib_ for each marker, and also cumulative P_ID_ and P_ID-sib_ for each multilocus genotype were calculated with GIMLET v.1.3.3 [47]. We considered that the risk of two individuals sharing the same genotype was negligible if P_ID-sib_ (more conservative than P_ID_) was lower than 0.01, as recommended for our population size estimates [51]. Then we estimated the minimum number of genotyped loci required to unequivocally individualize the samples, and discarded those samples not reaching the threshold.

For final individual identification results, we used the Difference in Capture History test (DCH) and the Examining-Bimodality test (EB) implemented in DROPOUT [49], using recommended values for P_ID_ and P_ID-sib_ [50] (see below). DCH examines if the rate of adding new individuals by adding more loci exceeds that expected just by increasing resolution, also a typical sign of a likely genotyping error. EB test looks for an over-abundance of genotypes observed only once, a typical sign of errors arisen during the genotyping process.

To reduce overestimation bias due to genotyping errors, we checked all pairs of samples differing in one or two alleles at one locus, or in two alleles at one or two loci. We evaluated consensus genotypes of these pairs of samples with a ‘matching approach’ [52]: for each pair, we identified the alleles that matched, and determined the probability of obtaining that particular set of matches by chance from two different capercaillie. If this probability is lower than P_ID-sib_ (see below), then we assumed that the samples probably came from the same individual. Otherwise, we kept the original consensus and considered they came from different capercaillies.

The reliability of each multilocus genotype obtained, was determined using RELIOTYPE [53]. Only scores with above 95% reliability were considered ‘acceptable’ without further analysis. Samples with lower scores were evaluated using information from sex primers and dropping location in the field, following the approach proposed by [54], adapted for our dataset and species: when the ‘unacceptable’ sample genotype corresponded to a recapture, i.e. when the genotype was identical to genotypes of other droppings, we considered (1) whether there was consistency of sex determination between the unacceptable sample and the other droppings belonging to the same genotype (sex agreement), (2) whether the dropping with the unacceptable genotype was collected in the same display area as another dropping with identical genotype (display area agreement) and (3) if the dropping with the unacceptable genotype was collected within a distance shorter than the maximum distance recorded in our dataset between samples with the same genotype (distance agreement). If the dropping in question passed two of the three agreement tests, it was recoded as ‘acceptable’ and kept in the database for further analysis. Otherwise, the sample was discarded.

### Minimum and estimated population size (N_min_, N_c_)

The total number of unique allelic combinations represents the minimum number of individuals inhabiting the area (minimum population size, *N_min_*), and provides a first approximation to the actual size of the population. *N_min_* was calculated using the ‘regroup genotypes’ function, implemented in GIMLET v.1.3.3 [47] and checked using DROPOUT [49] to verify whether genotyping errors were reduced to a non-significant level removing the risk of ‘shadow effects’[50].

From genotyped samples, capture-mark-recapture (CMR) estimates specifically developed for DNA-based recaptures can be obtained [29]. Unlike standard CMR studies, in DNA-based approaches an individual can be captured more than once per session (i.e. can be detected in more than one sample). Also, specific methods can estimate abundance from a single sampling session as long as individuals are sampled sufficiently to estimate recapture probabilities [55], [56]. To estimate census population size *N_c_*, we used a method based on genetic tagging [57] implemented in CAPWIRE software [55]. Each identified individual was treated as a ‘mark’ (initial capture), and a ‘recapture’ was recorded whenever an identical genotype was found in another independent DNA sample [29], [50]. To reduce over-sampling of a particular individual, if two samples, sharing the same XY coordinates and considered *a priori* as independent based on appearance, corresponded to identical genotypes, only one of them was included in CMR estimates. From the 132 reliable genotypes (see Results), eight additional samples were excluded from CMR estimates following this procedure, so that final number of samples considered for CMR estimates was 124. We also estimated *N_c_* for males and females separately (*N_c males_* and *N_c females_* respectively).

Our samples were relatively fresh and were collected during two months of intensive sampling. This sampling period was short enough to approximate the assumption of a closed population. The method accounts for capture heterogeneity using a likelihood ratio test to choose between two options: the even capture probability model (ECM) considers that every individual is equally likely to be captured, while the two innate rates model (TIRM) considers the population as a mixture of individuals that differ in capture probabilities, and is based on the simplest of those mixture models, with two groups of individuals with distinct capture probabilities [55].

We did not consider spatially explicit capture–recapture methods (SECR) because the lekking behavior of capercaillie in the breeding season does not fulfill the assumptions of independent spatial distribution and occupation of home ranges [58].

### Genetic effective population size (N_e gen_)

We estimated the genetic effective population size (*N_e gen_*) in the study area (i.e. effective population size as a consequence of population dynamics reaching back a few generations [59]). In addition, we estimated also *N_e gen_* in a subset of samples corresponding to Muniellos reserve, an area well represented in the dataset. We used two different approaches, specifically designed for single sample sessions, to estimate *N_e gen_*. First, we used a method based on the random linkage disequilibrium, and implemented in the software LDNe 1.31 [60]. This method assumes that only genetic drift - not mutation, selection or migration - is responsible for the signal in the data. Rare alleles incorrectly increase *N_e gen_* estimates, although this bias can be corrected by filtering out alleles below a given threshold of occurrence (*P_crit_*). We used *P_crit_* of 0.02 and 0.045 for the whole study area and Muniellos subset, respectively, based on sample size (i.e. number of individuals) [26], and we run the program under random mating reproductive strategy. We chose a jacknife procedure to get the 95% confidence intervals. We examined the effects of sample size (i.e. number of individuals) on *N_e gen_* by subsampling from 10 samples to total sample size via bootstrap re-sampling (100 iterations per sample size). We estimated *N_e gen_* at each sample size as the mean value obtained of 100 iterations. Since spatial distribution of samples can influence *N_e_* estimates [28], and our aim was to obtain a representative value for the whole study area, bootstraping was performed considering a stratified subsampling based in the five subzones of our study area, so that a proportional amount of samples from each area are included in each bootstrap.

We also estimated *N_e gen_* using the Approximate Bayesian Computation method (ABC) implemented in ONeSAMP 1.2. [61], which can increase accuracy and precision [33]. The user must provide presumed lower and upper bounds for *N_e gen_*. Taking into account that *N_e gen_* is usually much lower than census size, we used 2–50 as lower and upper bounds, respectively, for the whole study area (2–20 for Muniellos).

ONeSAMP cannot process multiple missing data per sample. Therefore we used the most complete subset of individuals correctly genotyped for these estimates (48 and 15 for the whole area and Muniellos, respectively). We used LDNe estimates to calculate the ratio *N_e gen_*: *N_c_*, so that both parameters are derived from the same samples (i.e. from the same individuals).

### Genetic tagging

We estimated maximum distances between recaptures of genetically-tagged individuals. To put those distances in context, we also calculated distances among display areas, as indication of the potential maximum distances for recaptures in the study area.

## Results

### Reliability of DNA genotyping

We obtained DNA from 212 samples (79% extraction success), and got consensus genotypes from 134 samples (63.2%, amplification success): in 128 samples consensus were reached with ≥7 microsatellite loci, and in 6 additional samples consensus were reached with only 5–6 loci; the latter included unique allele combinations and could be unequivocally individualized.

The number of alleles per microsatellite locus ranged between 3 and 6, and allele size ranged 121–234 (Table 2). The proportion of scoring errors across PCRs was 0.02, both for dropout and false allele (GIMLET 1.3.3). Mean error rate across loci was 0.03, dropout showing the highest probability (highest values found in TUT3, 0.15); mean rate for false alleles was 0.02 (GIMLET 1.3.3). Three loci (TUD4, BG10, TUT1) were not in Hardy-Weinberg equilibrium (GENEPOP 4.2, Table 2). We found no evidence for large allele dropout or scoring errors due to stuttering. The presence of null alleles was suggested for loci TUD5, TUT1 and BG10 (MICROCHECKER 2.2).

**Table 2:** Genetic parameters of Cantabrian capercaillie in the study area.

Locus	bp	N	A	He	Ho	P (HW)	PID	∏ PID	PID-sib	∏ PID-sib
TUD4	134–166	46	3	0.51	0.79	<0.0001	0.357	0.357	0.586	0.586
TUD2	180–190	52	3	0.49	0.44	0.708	0.303	0.108	0.585	0.343
TTD6	121–137	43	4	0.59	0.51	0.086	0.216	0.023	0.513	0.176
TUT3	156–164	46	3	0.60	0.51	0.555	0.230	0.005	0.509	0.089
BG15	140–156	52	5	0.62	0.63	0.938	0.204	0.001	0.491	0.044
TTD2	169–179	52	4	0.59	0.51	0.379	0.191	<0.001	0.480	0.021
BG10	204–220	37	5	0.69	0.42	0.000	0.147	<0.001	0.444	0.009
TUT1	214–234	43	5	0.70	0.40	0.004	0.133	<0.001	0.435	0.004
TUD5	180–194	51	6	0.72	0.68	0.805	0.098	<0.001	0.418	0.002
Mean		46.3	3.97	0.62	0.55					
SD		5.0	1.03	0.08	0.12					

Observed allele size (number of base pairs, bp), number of individuals correctly amplified (N), observed number of alleles per locus (A), estimated heterozygosity (H_e_), observed heterozygosity (H_o_), P-value for deviations from Hardy-Weinberg equilibrium (P(HW), α = 0.005), probability of identity (P_ID_), probability of identity for siblings (P_ID-sib_), and cumulative probabilities (∏ P_ID_ and ∏ P_ID-sib_) are shown for each marker. Means and standard deviations (SD) for the pool or markers are given for N, A, H_e_ and H_o_. N samples  = 134; N individuals  = 56; H_e_, H_o_, P (HW), P_ID_ and P_ID-sib_ were calculated using only one sample per individual. Microsatellites are ordered from least to most informative.

**Figure 2:**
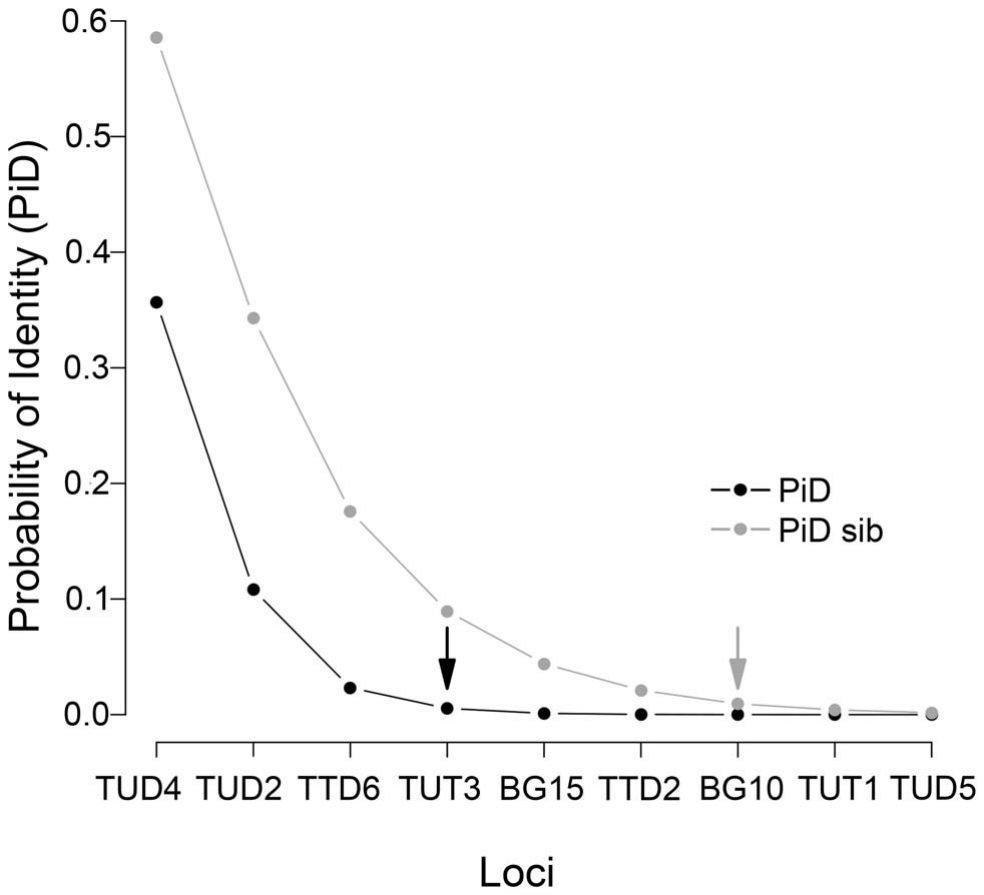
Multilocus probability of identity for unrelated (P_ID_) and sibling (P_ID-sib_) capercaillies. Probabilities were estimated with all the identified individuals (N = 56). Microsatellites are ordered from least to most informative. Arrows indicate the first value of P_ID_ and P_ID-sib_ below 0.01.

P_ID_ calculations showed that the power of the loci used to discriminate between individuals was high. The probability that two related individuals shared the same genotype for the nine loci used (cumulative P_ID-sib_) was 0.002 (Table 2, Figure 2). We followed a conservative threshold of the number of loci necessary to distinguish individuals (≥7 loci unambiguously genotyped, cumulative P_ID-sib_ <0.01; [50], [51].

When using a 7-loci consensus genotype as N_Lbase_ for DHC test (DROPOUT 2.3.1), results indicated that the contribution of each locus to the number of new individuals was well within the expected confidence intervals. We obtained the same results considering only those samples without missing values. We did not find any sign of bimodality (Bimodality test, DROPOUT), suggesting that there is not an over-abundance of genotypes observed only once, and discarding this potential source of error. One sample from the 134 consensus genotypes was discarded during the ‘matching approach’.

Average reliability of the consensus genotypes estimated with RELIOTYPE was 97.5%. Only 11 (8.9%) of our 133 samples were <95% reliable and therefore scored as ‘unacceptable’. Mean reliability value of these unacceptable samples was 75.03±0.05 SE. Nine of these samples had genotypes corresponding to recaptures (i.e. there were more samples with identical genotypes), and only 2 of them corresponded to unique genotypes. We applied the sex, display area and distance agreement tests to the 9 unacceptable samples corresponding to recaptures, and all them passed at least two of the three agreement tests. The two unacceptable unique genotypes could not be tested because they were found only once. One of them corresponded to the only sample found in a certain lek, and minimum distance to any other sample (5960 m) was larger than maximum distance recorded in our dataset between samples with the same genotype (3355 m); therefore it was kept in the database. The other unacceptable unique genotype was excluded from the database for further analyses, reducing to 132 the number of reliable consensus genotypes.

### Minimum and estimated population size (N_min_, N_c_), and sex ratio

We identified 56 different genotypes/individuals, which represent the minimum number of capercaillies (*N_min_*) in the study area. We detected 22 individuals more than once. The average number of observations per sampled individual was 2.21 (range 1–12), which should provide reliable estimates for a population ≤100 individuals [55].

Population size estimate *N_c_* (CAPWIRE, two innate rates model - TIRM) was 93 individuals in the study area (95% CI: 70–116). We estimated *N_c_* = 19 individuals (95% CI: 15–26, average recapture  = 3.53) in Muniellos. We were able to assign sex to 91% of the individuals, and found 19 females (*N_min female_*) and 32 males (*N_min male_*). Estimated *N_c female_* was 28 (95% CI: 20–41, average recapture  = 1.63) and *N_c male_* was 44 (95% CI: 33–56, average recapture  = 2.75). Sex ratio, estimated as *N_c female_: N_c male_* was 1∶1.57.

### Genetic effective population size (N_e gen_)

LDNe and ONeSAMP yielded similar estimates of *N_e gen_* for the whole study area: 35.5 (95% CI, 21.6 – 67.7) and 32.8 (95% CI 25.5 – 46.1), respectively. For Muniellos, LDNe yielded a slightly lower value (6.8, 95% CI: 2.5 – 22.5) than ONeSAMP (9.8, 95% CI: 8.1 – 12.3).

Different *P_crit_* did not alter LDNe estimates (Table S1). The results using 6 and 9 microsatellite loci in ONeSAMP were similar, but results in LDNe were less consistent and showed larger CIs when considering just 6 loci. Different priors did not yield very different estimates in ONeSAMP (Table S2).

**Figure 3:**
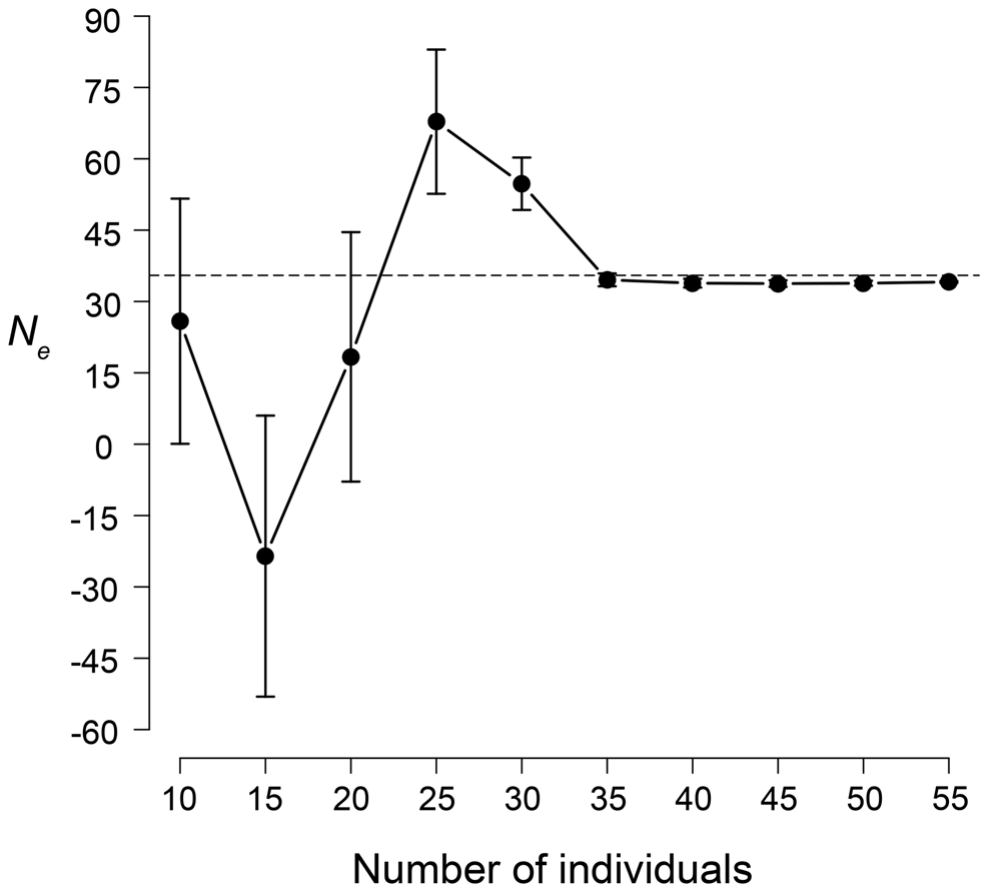
Estimates of *N_e gen_* from LDNe method vs. number of identified individuals. Each data point represents the arithmetic mean (± SE) of 100 bootstrapping iterations, stratified among the five zones in our study area.

Subsampling indicated that *N_e gen_* estimates for the whole area were relatively insensitive to sample sizes (i.e. number of individuals) larger than 35 (Figure 3). Standard error also gets substantially lower above this sample size. It is noticeable that for a quite small sample size (N = 15), *N_e gen_* values for Muniellos were relatively consistent, irrespective of the approach and parameters considered, while for the whole area a similar sample size was clearly below the minimum threshold to get a reliable estimate.

The ratio *N_e_*_ gen_: *N_c_* was 0.38 for the whole area and 0.36 for Muniellos.

### Genetic tagging

**Figure 4:**
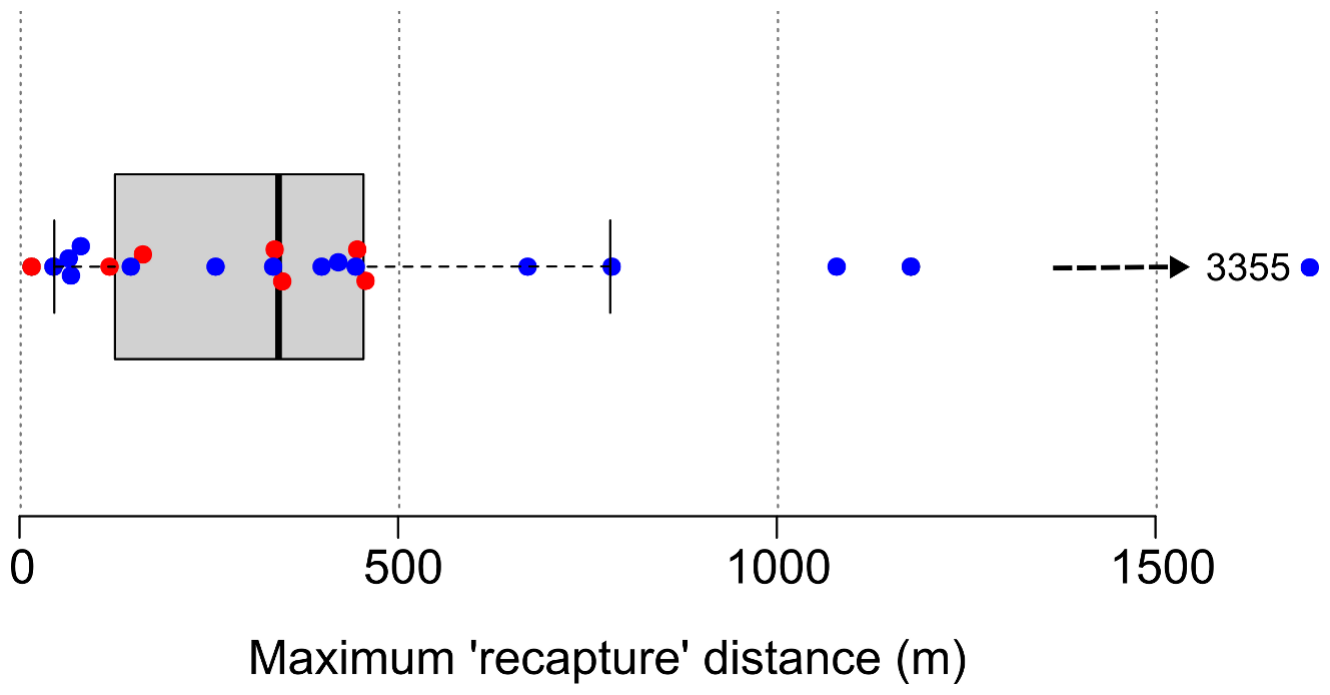
Maximum distances between recaptures. Box-plot shows the distribution of maximum distances (m) between genetic recaptures. Center lines show the median; box limits indicate the 25th and 75th percentiles, and whiskers extend 1.5 times the interquartile range. Red and blue dots indicate females and males, respectively (n = 22); the arrow and value at the right end of the plot indicate a male outlier beyond the axis range.

Movements were estimated from genetic recaptures in 22 individuals, 7 females and 15 males. Three additional individuals were also recaptured (and included in CMR analyses), but movements were not calculated for them because their location was not obtained with GPS. Median planimetric distances recorded between recaptures were 337 m for females and 399 m for males, although the latter showed a distribution skewed towards longer distances (Figure 4). Mean distance between contiguous, sampled display areas was 1362 m (±142 SE), and the maximum distance between sampled areas was 40,855 m. Three males were recaptured in more than one display area, although these recaptures corresponded to contiguous display areas.

## Discussion

Previous studies have reported that the Cantabrian subspecies of capercaillie is endangered, based on severe reduction of display area occupancy [4]. Our abundance estimates show that decline in display area occupancy is indeed associated with a very low population size, well within concern in terms of population viability (e.g. [62], [63]). We estimated that 93 individuals (*N_c_*, 70–116 95% CI) gathered for their spring courtship in 34 display areas (Figure 1), in an area that likely harbours most of the extant population.

### Sex ratio and descriptive genetic parameters

We found a higher proportion of males in the population (sex ratio 1∶1.6). The combination of a small population size and its inherent risk of extinction with a male-biased sex ratio in a polygynous species such as capercaillie, could constitute an additional threat [64] if sustained over generations. However, such result is not rare in wild birds, and is not necessarily associated to poor conservation status. Higher female mortality or differential behavior and ecology leading to male-biased sampling schemes could explain skewed sex ratios [65], although less intrusive surveys like ours may better approach actual sex ratios [66], [67]. We also obtained a lower recapture rate for females, which could indicate shorter or more discontinuous presence of the latter in display areas [14], [15], reducing in turn the odds of finding their droppings.

Despite the small number of extant individuals in the population, expected and observed heterozygosity levels and number of alleles (Table 2) were within the range of other, less threatened capercaillie populations [68]. Our results are also higher than previously reported values for Cantabrian capercaillie (H_e_ = 0.50 and A = 3.40) [69]. The latter discrepancy is likely due to substantial differences in scope and sampling area of both studies: we aimed at obtaining a higher resolution in a smaller area, where not only decline but also genetic isolation processes seemed to be less acute.

### Genetic effective population size

The ratio between genetic effective population size and census population size (*N_e gen_*: *N_c_*) was 0.38, suggesting that the proportion of the population contributing to reproduction is relatively large [24]. Our result is notably higher than the empirical average across many taxa, 0.1 [18], although that ratio seems largely context-dependent. Demographic factors such as fluctuation in population size, variance in family size, and unequal sex-ratio add large variability to *N_e_*: *N_c_* [22], [70]. Taxonomic group is also relevant, and published averages for birds (0.21) are closer to our result [18]. Perhaps more relevant, recent evidence showed that the contemporary *N_e gen_* of a population is sensitive to the geographic scale of the survey [28]. Thus, not only sample size but also the spatial distribution of samples can affect *N_e gen_* estimates (note that we obtained similar results for the whole study area and the subset of Muniellos, 0.38 and 0.36, respectively). In addition, since the population has been declining, our *N_e gen_*: *N_c_* ratio may be somewhat slightly inflated: the genetic effective size *N_e gen_* can be roughly related to the number of breeders in previous generations, whereas *N_c_* corresponds to present population size [71]. At any rate, evaluating *N_e gen_* and *N_e gen_: N_c_* ratios makes special sense in intra-specific comparisons and in population monitoring, following specific and comparable sampling designs.

### Genetic tagging

We detected mostly short movements of capercaillie within the breeding season (Figure 4), and just a few males recaptured between contiguous leks. Overall, breeding capercaillie tend to stick to one lek during display season [72]. However, in small fragmented capercaillie populations females could be forced to visit several leks to find a mate [73]. Our data, albeit limited in sample size, do not conform to the latter. It is also known that non-breeding capercaillies move more among leks than breeders [16], [73]. We cannot infer the age from our samples, yet detected inter-lek movements could be due to juvenile individuals. It would also be very interesting to use genetic tagging of this population in autumn, when dispersal of juveniles could perhaps change the picture offered by our spring data.

Despite its potential, genetic tagging has not often been used to estimate individual movements (but see [74]–[76]). There are undoubtedly caveats that prevent straightforward comparison between genetic tagging and directly recorded movements, essentially because the former does not really contact and follow the individuals. In addition, individual recaptures will usually be relatively low. However, genetic tagging does not require capturing the animals, which is often an issue in endangered populations. It could also complement radio tracking to get appropriate sample size when logistics are demanding or budget is tight.

### Influence of survey design on population estimates

Monitoring is important to evaluate the status of populations, to support management decisions, and eventually to evaluate the efficiency of those decisions. However, deficient monitoring programs can lead to wrong conclusions, even entailing risks for the targeted species (e.g. [77]). Surveys intended to estimate population size should be designed to include all sectors of the population [12], [13]. Besides, the design should take into account the natural history and behavioral peculiarities of the focal species. In this sense, capercaillie gather at display areas in spring, so it is important to know which proportion of known display areas were included in the survey. Our estimates included 71% of known lekking places in the study area. These results provide reference for future monitoring, or to establish comparisons with similar surveys elsewhere.

Sample size can also have a substantial effect on estimates of *N_e gen_* and *N_e gen_*: *N_c_* ratios, and could lead to inconsistencies or even absurd results if below a minimum threshold (Figure 3). This has been a mostly overlooked aspect, which could be partly responsible for the large variability of published in results of *N_e gen_*: *N_c_* ratio.

## Supporting Information

Table S1 **Estimated** ***N_e gen_*** **from LDNe.** Genetic effective population size (*N_e gen_*, 95% CI in parentheses), estimated for the whole study area and for Muniellos reserve. We show estimates using different minimum threshold frequencies (*P_crit_*) for an allele to be included in the estimates. We also show the estimates when including all microsatellite loci used in our study (9 loci), and excluding those that were not in Hardy-Weinberg equilibrium. Results for the whole area are shown using 48 individuals (only one missing value) and 56 individuals (up to two missing values). In bold, chosen estimates (see criteria in Methods), mentioned in the text.
(DOCX)

Table S2 **Estimated** ***N_e gen_*** **from ONeSAMP.** Genetic effective population size (Ne gen, 95% CI in parentheses), estimated for the whole study area (48 samples) and for Muniellos reserve (15 samples). We explored the variability in the mean estimate and range using different priors (minimum and maximum values estimated a priori for the effective population size). We also show the estimates when including all microsatellite loci used in our study (9 loci), and excluding those that were not in Hardy-Weinberg equilibrium. In all cases, we used samples with only one missing value. Bold font indicates estimates used in the text.
(DOCX)
