# Supplementary material for: Demographic Status and Genetic Tagging of Endangered Capercaillie in NW Spain
Source: PLoS One. 2014 Jun 13;9(6):e99799. doi: 10.1371/journal.pone.0099799 (PMC4057396; doi:10.1371/journal.pone.0099799)
Supplement: Table S1 — Estimated Ne gen from LDNe. Genetic effective population size (Ne gen, 95% CI in parentheses), estimated for the whole study area and for Muniellos reserve. We show estimates using different minimum threshold frequencies (Pcrit) for an allele to be included in the estimates. We also show the estimates when including all microsatellite loci used in our study (9 loci), and excluding those that were not in Hardy-Weinberg equilibrium. Results for the whole area are shown using 48 individuals (only one missing value) and 56 individuals (up to two missing values). In bold, chosen estimates (see criteria in Methods), mentioned in the text. (DOCX) [file pone.0099799.s001.docx]

S1 Table. Estimated *N_e gen_* from LDNe.

|  |  |  | *N_e gen_* (95% CI) | | | |
| --- | --- | --- | --- | --- | --- | --- |
|  | N samples | N loci | P_crit_ 0.05 | P_crit_ 0.045 | P_crit_ 0.02 | P_crit_ 0.01 |
| Whole study area | 48 | 9 | 24.3 (15.3-41.8) | 24.7 (15.7-42.0) | 31.4 (19.2-58.9) | 41.9 (23.9-95.5) |
|  | 48 | 6 | 43.1 (18.5-263.7) | 41.7 (18.1-223.9) | 52.1 (20.8-1063.9) | 47.6 (19.7-440.9) |
|  | 56 | 9 | 25.9 (16.5-44.0) | 25.3 (16.3-42.3) | 35.5 (21.6-67.7) | 45.3 (26.0-102.8) |
|  | 56 | 6 | 53.3 (22.0-552.0) | 52.8(22.0-485.0) | 65.6 (25.0-∞) | 55.6 (22.7-754.7) |
| Muniellos | 15 | 9 | 6.8 (2.5-22.5) | 6.8 (2.5-22.5) | 8.4 (2.7-32.7) | 8.4 (2.7-32.7) |
|  | 15 | 6 | 4 (1.6-23.4) | 4 (1.6-23.4) | 3.6 (1.6-20.7) | 3.6 (1.6-20.7) |

Genetic effective population size (*N_e gen_*, 95% CI in parentheses), estimated for the whole study area and for the subset of Muniellos reserve. We show estimates using different minimum threshold frequencies for an allele to be included in the estimates (*P_crit_*). We also show the estimates when including all microsatellite loci (9 loci), and excluding those that were not in Hardy-Weinberg equilibrium. Results for the whole area are shown using 48 individuals (only one missing value) and 56 individuals (up to two missing values). Bold font indicates estimates used in the text.
