# Supplementary material for: Demographic Status and Genetic Tagging of Endangered Capercaillie in NW Spain
Source: PLoS One. 2014 Jun 13;9(6):e99799. doi: 10.1371/journal.pone.0099799 (PMC4057396; doi:10.1371/journal.pone.0099799)
Supplement: Table S2 — Estimated Ne gen from ONeSAMP. Genetic effective population size (Ne gen, 95% CI in parentheses), estimated for the whole study area (48 samples) and for Muniellos reserve (15 samples). We explored the variability in the mean estimate and range using different priors (minimum and maximum values estimated a priori for the effective population size). We also show the estimates when including all microsatellite loci used in our study (9 loci), and excluding those that were not in Hardy-Weinberg equilibrium. In all cases, we used samples with only one missing value. Bold font indicates estimates used in the text. (DOCX) [file pone.0099799.s002.docx]

S2 Table. Estimated *N_e gen_* from ONeSAMP.

|  | **N samples** | **N loci** | **Priors (min-max)** | ***N_e gen_* (95% CI)** |
| --- | --- | --- | --- | --- |
| **Whole study area** | 48 | 9 | 2-100 | 27.2 (21.8-37.9) |
|  | 48 | 6 | 2-100 | 30.1 (23.7-45.4) |
|  | 48 | 9 | 2-200 | 23.7 (18.8-36.1) |
|  | 48 | 6 | 2-200 | 26.9 (20.9-49.9) |
|  | 48 | 9 | 2-50 | 25.8 (20.9-36.1) |
|  | 48 | 6 | 2-50 | **32.8 (25.5-46.1)** |
| **Muniellos** | 15 | 9 | 2-50 | 12.9 (10.5-17.8) |
|  | 15 | 6 | 2-50 | 12.4 (9.7-17.5) |
|  | 15 | 9 | 2-20 | 13.1 (10.9-16.9) |
|  | 15 | 6 | 2-20 | **9.8 (8.1-12.3)** |

Genetic effective population size (*N_e gen_*, 95% CI in parentheses), estimated for the whole study area (48 samples) and for the subset of Muniellos reserve (15 samples). We explored the variability in the mean estimate and range using different priors (minimum and maximum values estimated a priori for the effective population size). We also show the estimates when including all microsatellite loci used in our study (9 loci), and excluding those that were not in Hardy-Weinberg equilibrium. In all cases, we used samples with only one missing value. Bold font indicates estimates used in the text.
